# Recent Progress of Adsorptive Ultrafiltration Membranes in Water Treatment—A Mini Review

**DOI:** 10.3390/membranes12050519

**Published:** 2022-05-13

**Authors:** Tong Yu, Jing Zhou, Feng Liu, Bao-Ming Xu, Yong Pan

**Affiliations:** 1 Hubei Provincial Key Laboratory of Green Materials for Light Industry, Hubei University of Technology, Wuhan 430068, China; yt15549070413@126.com (T.Y.); kugougou1314@163.com (F.L.); xubaoming897@163.com (B.-M.X.); 2 Collaborative Innovation Center of Green Light Weight Materials and Processing, Hubei University of Technology, Wuhan 430068, China; 3 Patent Examination Cooperation Hubei Center of the Patent Office, CNIPA, Wuhan 430075, China; jingzou0430@163.com

**Keywords:** adsorptive ultrafiltration membrane, water reuse, harmful cations, small-molecule organics, mechanism

## Abstract

Adsorptive ultrafiltration mixed matrix membranes (MMMs) are a new strategy, developed in recent years, to remove harmful cations and small-molecule organics from wastewater and drinking water, which achieve ultrafiltration and adsorption functions in one unit and are considered to be among the promising technologies that have exhibited efficiency and competence in water reuse. This mini review concerns the research progress of adsorptive ultrafiltration MMMs for removing heavy metal ions and small-molecule organics. We firstly introduce the types and classifications of adsorptive ultrafiltration MMMs (their classifications can be established based on the type of the adsorbent used). Furthermore, we discuss the removal mechanism of adsorptive ultrafiltration MMMs, as well as summarizing the main fabrication techniques for adsorptive ultrafiltration membranes. In addition, we identified some of the issues and challenges of the practical application for adsorptive ultrafiltration.

## 1. Introduction

Water is the source of life. Without water, there is no future [1]. There are plenty of water resources on earth, however, due to low per capita freshwater resources, unbalanced temporal and spatial distribution of water resources, and poor utilization efficiency of water resources, human beings have a serious situation of insufficient or even a shortage of water resources [2,3]. At the same time, with the rapid development of some industries, such as the electroplating industry, mining industry, battery industry, paper industry, and the agricultural pharmaceutical industry, more and more heavy metals (such as zinc, copper, nickel, mercury, cadmium, lead, and chromium, etc.) are directly or indirectly discharged into the environment. In addition, small-molecule organics (such as bisphenol A, polychlorinated biphenyls, industrial synthetic substances, phthalate lipids, acetochlor, and other pesticide substances) are harmful chemicals that are also released into the environment due to human production and life. These trace heavy metals and small-molecule organics cause irreversible damage to the ecological environment and to human beings [4,5,6]. Water resources are necessary for human development, especially the safety of drinking water, which is directly related to people’s life, health, and safety. Therefore, it is very important to remove heavy metals and small-molecule organics from wastewater and drinking water.

Membrane-based water treatment processes have great potential in sustainable water purification and provide a viable avenue for producing potable water due to their high flux, good performance, and low negative effects. In the field of water treatment, although reverse osmosis and nanofiltration can remove small-molecular organics and heavy metal ions, it is difficult to apply them to urban water supply treatment on a large scale because of the high operating pressure and high energy consumption [7]. Ultrafiltration has the characteristics of good treatment effect, low energy consumption, high reliability, and stable operation. It can almost completely remove protozoa, bacteria, and some viruses from water. The commonly used ultrafiltration membrane manufacturing materials in the market are polysulfone (PSF), polyacrylonitrile (Pan), polyvinylidene fluoride (PVDF), and polyethersulfone (PES). In addition, there are polytetrafluoroethylene (PTFE), polyvinyl chloride (PVC), polypropylene (PP), polyethylene (PE), etc., but these membranes have poor pollution resistance and cannot intercept some small-molecular organics and heavy metal ions.

Adsorbents are promising materials for capturing pollutants, such as low-molecular organics and heavy metal ions, because of their abundant sorption sites, large surface area, and fast adsorption kinetics [8,9,10,11,12,13,14]. However, adsorbents are usually synthesized in the form of powders, which give rise to some problems in the separation and regeneration processes [15,16] and potential safety issues may occur due to leaching into water bodies [17,18,19]. Furthermore, it is difficult to use particles directly to retain macro-molecules and particulates. Therefore, there is an urgent need to develop the next generation ultrafiltration membrane technology with both interception and adsorption performance in order to achieve economic and efficient water treatment.

Adsorptive ultrafiltration MMMs appear when polymers and adsorbents with adsorption capacity are fixed in the membrane instead of being added into the wastewater. It is still a challenge to combine the advantages of adsorbent and ultrafiltration membranes successfully and to overcome their respective shortcomings in water treatment.

In recent years, a large number of studies have focused on adsorptive ultrafiltration MMMs due to their multiple advantages. This mini review aims to give a critical review of the current developments of adsorptive ultrafiltration MMMs for water treatment. The type and classification of adsorptive ultrafiltration MMMs will also be summarized here. Particular emphasis will be given to the summary and analysis of the different adsorptive ultrafiltration MMMs and their mechanisms. In addition, the future trends and challenges for the development of adsorptive ultrafiltration MMMs will also be given.

## 2. Classifications of Adsorptive Ultrafiltration Membrane

Ultrafiltration membranes with adsorption function have been reported in literature. Based on the type of adsorbent added to the membrane, adsorption ultrafiltration MMMs can be divided into the following four categories: inorganic filler, organic filler, biomaterial, and mixed filler membrane. Table 1 lists the removal results of these four adsorptive ultrafiltration MMMs.

### 2.1. Inorganic Filler-Based MMMs

These advanced adsorptive ultrafiltration membranes contain inorganic fillers, such as Al_2_O_3_ [20], ZnO [21], MWCNT [22], carbon nanotubes [23], graphene oxide [24], zeolite [25], and activated carbon [26]. These inorganic fillers significantly improve the adsorption performance of the membrane. For example, copper ion removal efficiency improved from 25% to 60% just by adding small amounts of Al_2_O_3_ nanoparticles (≤1.0 wt.%) into polyethersulfone (PES) membranes [20]. Shah and Murthy [22] added functionalized multi-walled carbon nanotubes (MWCNT) into polysulfone (PSF) membranes through the phase inversion method, using DMF as a solvent and water with isopropanol as a coagulant. The functionalized MWCNT/PSF composite membranes displayed 94.2% removal for Cr(VI) and 78.2% removal for Cd(II), however, the unblended plain polysulfone membranes only displayed 10.2% removal for Cr(VI) and 9.9% removal for Cd(II), respectively. In addition, using zeolite nanoparticles impregnated polysulfone membranes for the removal of heavy metals in wastewater [25]. After 60 min of filtration at a transmembrane pressure of one bar, the maximum adsorption capacities of the mixed membrane for lead and nickel ions were 682 and 122 mg/g, respectively. The addition of hydrophilic inorganic fillers into the polymeric membranes mainly resulted in a significant improvement of water flux, which was attributed to an increase in hydrophilic properties that decreased the contact angle, coupled with greater surface roughness and overall porosity [27,28,29]. Using this type of adsorptive ultrafiltration MMM not only improves the flux and rejection, but also prevents membrane fouling due to the increased hydrophilicity [30].

### 2.2. Organic Filler-Based MMMs

In this case, organic fillers, such as polyvinyl tetrazole (PVT) [31], polyaniline (PANI) [32], hyperbranched polyester [33], and 2-aminobenzothiazole [34], are added by the methods of blending and phase inversion. Kumar et al. [31] manufactured polyvinyl tetrazole−co−polyacrylonitrile (PVT−co−PAN) membranes by nonsolvent induced phase separation (NIPS). After adding the PVT segment, the prepared adsorption ultrafiltration MMMs became more negatively charged and hydrophilic due to the existence of -NH- functional groups. The PVT segment in the membrane is the main binding site for adsorbing Cu (II) ions in aqueous solution, and the adsorption capacity can reach 44.3 mg g^−1^, which is higher than the other membranes reported in the literature. In addition, Ding et al. [32] prepared a charged UF membrane composite (PANI/PVDF) that was regulated via an electrochemically reversible control in portions of amine (-N^+^=:)/imine (-NH-) functional groups of PANI. The permeability of treated water and rejection ratios of Congo red anion on charged PANI/PVDF, compared with pristine a PVDF membrane, increased from 19.6 to a maximum of 183.3 Lm^−^^2^ h^−1^ bar^−1^ and from 3.4% to 74%, respectively. Moreover, through electrochemical regulation, the rejection ratio of Congo red on PANI/PVDF reached up to 93%. In addition, hyperbranched polyester that was cross-linked with PVC was used to form a PVC–UF composite membrane, which has a high permeate flux of 237.6 L m^−^^2^ h^−^^1^ and a good sunset yellow anion rejection rate of 96.4% at 0.4 Mpa [33]. This type of membrane is preferred over the inorganics as they have more functional groups, which makes them more adaptable and capable to attach cations and small-molecule organics to the substrate through molecular interactions.

### 2.3. Biomaterial-Based MMMs

Recently, because biomaterial-based adsorbents have the advantages of eco-friendliness, accessibility, and low cost (or even free of expense), research studies have particularly focused on the biomaterial-based adsorbents that stem from plant wastes, such as hulls, tea leaves, fruit peels, plant seeds, and so on. [35]. Usually, plant wastes, including some groups of COOH, OH, or phenolics, can provide charge interaction and hydrogen-bonding interaction with cations and small-molecule organics [35,36]. For instance, Aquaporin Z was incorporated into a triblock copolymer with symmetric poly-(2-methyloxazoline)-poly-(dimethylsiloxane)-poly-(2-methyloxazoline) (PMOXA15-PDMS110-PMOXA15) vesicles and the performance of the adsorptive ultrafiltration MMMs were investigated for the removal of urea, glucose, glycerol, and salt from water. The results showed that these solutes were completely rejected [37]. Lin et al. [38] reported an adsorptive ultrafiltration MMM using plant waste (including banana peel, tea waste, and shaddock peel) as biofiller in polyethersulfone and evaluated the removal performance of cationic dyes from water. The rejection of dye molecules reached up to 95%.

### 2.4. Hybrid Filler-Based MMMs

Hybrid filler-based MMMs contain two organic–inorganic adsorbents (independently or in composite) and metal–organic framework (MOF) materials are added to the polymer solutions, which represent the latest adsorptive ultrafiltration MMMs technology [39,40,41,42]. For example, Daraei et al. [39] added iron (II, III) oxide and polyaniline into a PSF matrix, with which the removal of Cu (II) can be 85% from water, and this membrane can be reused after four cycles with only about 3% decrease in the rejection capability. In the study of Parsamanesh et al. [40], polyethersulfone-based MMMs incorporated with citric acid–amylose-decorated multiwall carbon nanotubes (Am–MWCNTs–CA) were fabricated. The humic acid removal capability of the prepared membranes was also calculated to be as high as 97.4% for the membrane that was embedded with 0.5 *w*/*v*% Am–MWCNTs–CA. Furthermore, Zhang et al. [41] presented the MIL-PVDF multifunctional ultrafiltration membrane with ultra-high MIL loading through a new method of predispersion and thermally induced phase separation in acetone. Compared with the traditional mixed ultrafiltration membrane, the effective treatment volume of 67-MIL-PVDF membrane increased by nine times, and the MB removal rate was more than 75%. In addition, Zhang et al. [42] prepared a new MOF-based hybrid ultrafiltration MMM (PAA/ZIF-8/PVDF membrane), which is superior to other adsorption materials and has the first and highest nickel ion (Ni (II)) adsorption capacity of 219.09 mg/g in high salinity wastewater.

**Table 1:** Summary for fabrication techniques.

Type of MMMs	Membrane	Adsorbent	Pollutants	Rejection	Ref.
Inorganic filler-based MMMs	PES	Al_2_O_3_	Cu^2+^	60.0%	[20]
	PVDF	ZnO	Cu^2+^	83.3%	[21]
	PSf	MWNTs	Cr^5+^	94.2%	[22]
	PVC	CNT	Fe^2+^	95.1%	[23]
	PES	GO	Pb^2+^	98.0%	[24]
	PSF	NaX	Pb^2+^	91.0%	[25]
	PES	Carbonaceous materials	Cu^2+^	79.1%	[26]
Organic filler-based MMMs	PAN	PVT	Cu^2+^ Pb^2+^	98.5% 51.0%	[31]
	PVDF	PANI	Congo red	74.0%	[32]
	PVC	Hyperbranched polyester	Sunset yellow	96.4%	[33]
	PVDF	2-aminobenzothiazole	Cr^5+^	82.1%	[34]
Biomaterial-based MMMs	PMOXA15-PDMS110- PMOXA15	Aquaporin Z	Urea, glucose, glycerol	100%	[37]
	PES	Banana peel, tea waste, and shaddock peel	Methylene blue Methyl violet 2B	95.0% 96.0%	[38]
Hybrid filler-based MMMs	PES	Iron (II, III) oxide and polyaniline	Cu^2+^	85.0%	[39]
	PES	Citric acid–amylose-decorated multiwall carbon nanotubes	Humic acid	97.4%	[40]
	PVDF	MIL	MB	75.0%	[41]
	PVDF	PAA/ZIF-8	Ni^2+^	99.0%	[42]

## 3. Mechanisms of Adsorptive Ultrafiltration MMMs

**Figure 1:**
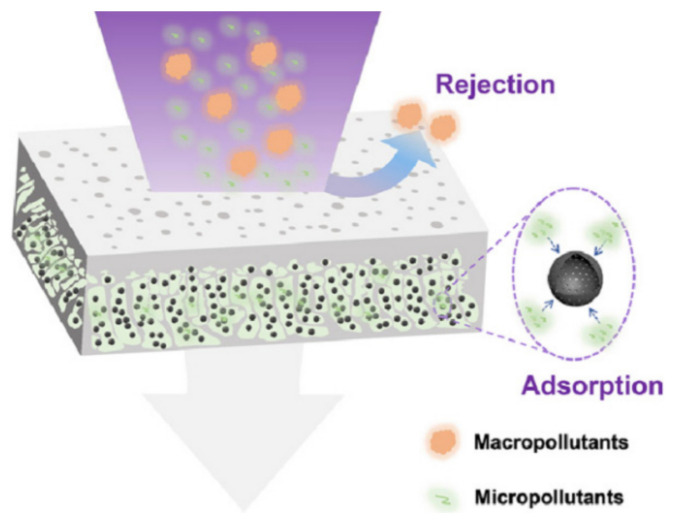
The purification process for multiple pollutants polluted water by DFUF membrane. Reprinted/adapted with permission from Ref. [43]. Copyright © 2020 Elsevier.

Most commonly, the removal of heavy metal ions and organic molecules from wastewater by adsorptive ultrafiltration MMMs is based on the rejection-adsorption mechanism [43]. The selective removal of such pollutants by the adsorptive ultrafiltration MMMs is demonstrated in Figure 1. When wastewater contains heavy metal ions and organic molecules contact the diffuse layer of the adsorptive ultrafiltration membrane, those molecules with sizes larger than the adsorptive ultrafiltration membrane’s pore size are rejected due to molecular sieving. In addition, a part of the small molecules and ions are rejected via repulsive force with the charged membrane surface, another part of small molecules and ions can pass through the diffuse layer and reach to the stern layer, then will undergo adsorption in the stern layer by the adsorbent material. The adsorption mechanism of adsorptive ultrafiltration MMMs usually includes electrostatic interaction, hydrogen-bond interaction, and complexation. The adsorbent in the adsorptive ultrafiltration MMMs may contain reactive functional groups (e.g., -NH_2_ or -COOH), which can interact with heavy metal ions and small-molecule organics by electrostatic interaction, hydrogen-bond interaction, or complexation [44]. For example, Rowley et al. [45] synthesized polyethersulfone (PES) nanocomposite membranes using surface modified Fe_3_O_4_ nanoparticles (NPS) for the removal of arsenic from water. The arsenic pollutants were removed by adsorption of the polymer membrane because of the functional groups of iron oxide (Fe_3_O_4_) microspheres. The small size arsenic pollutants passed through the diffusion layer and reached the stern layer, then reacted with Fe_3_O_4_ through adsorption to form a tight internal spherical complex. For the PES membrane, with three wt.% of Fe_3_O_4_ NP, the maximum rejection rate of arsenic was 76%, and the maximum arsenic equilibrium adsorption capacity was 14.6 mg/g.

## 4. Preparation Techniques for Adsorptive Ultrafiltration Membrane

**Figure 2:**
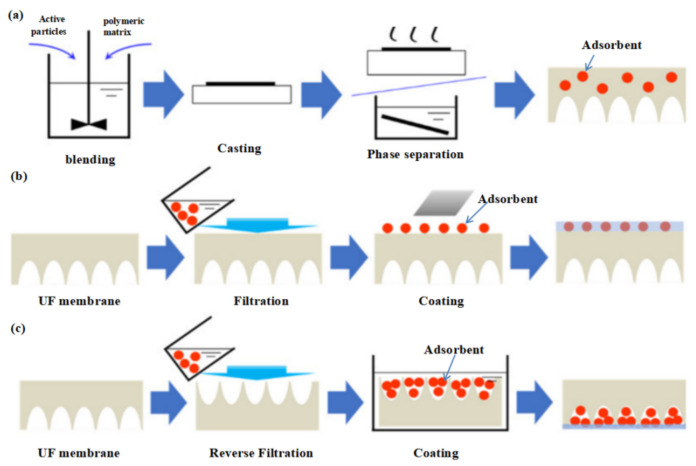
Schematics of some typical methods for the preparation of multifunctional ultrafiltration membranes: (**a**) blending, (**b**) surface coating, and (**c**) reverse filtration. Reprinted/adapted with permission from Ref. [50]. Copyright © 2020 Elsevier.

The preparation methods of adsorption ultrafiltration membranes mainly include blending, surface coating, and the reverse filtration method, which are conventional methods used to prepare adsorption ultrafiltration membranes. Figure 2 summarizes the schematic diagram of typical preparation methods of these multifunctional ultrafiltration membranes.

### 4.1. Blending

In this case, adsorptive ultrafiltration MMMs were prepared through incorporating adsorbents into a polymeric matrix in an organic solvent, then the casting solution is solidified via phase separation. Zhang et al. [21] blended ZnO nanoparticles with PVDF solution and then cast films. The PVDF/ZnO adsorptive ultrafiltration MMMs have a uniform structure that the ZnO nanoparticles were incorporated into via the pores and onto the surface of PVDF, which improved the hydrophilicity, permeability, and antifouling performance of MMMs, compared with the pristine PVDF films. In addition, Wang et al. [34] prepared a new modified ultrafiltration membrane by blending polyvinylidene fluoride (PVDF) with 2-aminobenzothiazole through phase transformation. Compared with the neat PVDF membrane, the contact angle of the modified PVDF/2-aminobenzothiazole ultrafiltration membrane decreased from 79.3° to 76.1°, the pure water flux increased from 160 L/m^2^·h to 231.27 L/m^2^·h, and the adsorption capacity of chromium ion increased from 85 µg/cm^2^ to 157.75 µg/cm^2^. The adsorption capacity of the PVDF/2-aminobenzothiazole ultrafiltration membrane for chromium ion is better than that of the traditional PVDF membrane. So far, blending active materials with the polymer substrate is the most typical method for the fabrication of adsorptive ultrafiltration MMMs. Adsorbent materials can be uniformly dispersed within the membrane via the blending method. The introduction of hydrophilic adsorbent materials can improve the surface property of the adsorptive ultrafiltration MMMs, thus not only enhancing the separation efficiency of the membrane but also pollution resistance. In addition, the blending preparation method is convenient for one-step membrane production.

### 4.2. Surface Coating

In general, surface coating is a three-step membrane production process. Firstly, fabricating the ultrafiltration membrane. Secondly, loading the adsorbent materials on the top surface of the as-synthesized ultrafiltration membrane through immersion or filtration. In addition, fixing adsorbent materials. For example, Li et al. [46] design a novel cationic metal–organic framework hybrid ultrafiltration polyvinylidene fluoride membrane (PVA/Cu-iMOFs/PVDF-0.05) using the surface-coating method and investigated its unique capture of aqueous perchlorate (ClO_4_^−^) at ppm-level. The results showed the ClO_4_^−^ removal ratio reached 99.6% over a wide pH range (3–10) and there was excellent long-term stability in the cross-flow filtration process. The membrane could be regenerated in acid solution, there is a negligible decrease in capacity for repeated use. Cetinkaya et al. [47] prepared graphene oxide (GO)-coated membranes, by coating graphene oxide onto the membranes using an air spray method, and investigated microfiltration, ultrafiltration, and nanofiltration membranes coated with GO and the efficiency of As(III) removal under 5 bar pressure were 98%, 100%, and 100%, respectively. Through this method, the adsorption material can be evenly dispersed on the top of the ultrafiltration membrane so as to improve the decontamination efficiency of the ultrafiltration membrane. In addition, the adsorption performance of the intercepting material can also be improved due to the ordered arrangement.

### 4.3. Reverse Filtration

The reverse filtration method is also a three-step membrane production process. Fabricating the ultrafiltration membrane firstly, and then filtrating the adsorbent materials from the bottom of the prepared ultrafiltration membranes and fixing the adsorbent materials with a material. Ren et al. [48] fabricated the separation layer and support layer of a polyacrylonitrile (Pan) UF membrane and modified it using polyethyleneimine (PEI), metal–organic framework (MOF), laccase, and polydopamine (PDA), which is termed as “three dimensional (3D) modification”. The LacPAN-MIL-101 membrane achieved a high BPA removal efficiency of 92% in one flow-through cycle under an ultra-low laccase dosage. Fang et al. [49] designed a novel ultrafiltration membrane using the reverse filtration method, through modifying the inner pore wall of the membrane with polydopamine (PDA) nanoparticles. Adsorptive ultrafiltration membrane showed an increase in the rejection rate of bovine serum albumin (BSA) (92.9%) and a sustainable pure water flux (166 L/m^2^ h). Meanwhile, the static adsorption capacities of the adsorption ultrafiltration membrane for Pb^2+^, Cd^2+^, and Cu^2+^ were 20.23 mg Pb/g, 17.01 mg Cd/g, and 10.42 mg Cu/g, respectively. Although reverse filtration has low requirements for the supporting ultrafiltration membrane compared with other methods, this method also is confronted with the problem of a complicated fabrication process and may suffer from particle loss, which might be more serious than in the surface-coating method. In addition, the membrane resistance to fouling was not enhanced as compared to other methods.

All in all, the blending of ultrafiltration membranes with hydrophilic nanoadsorbent materials is a simple and convenient method to endow them with antifouling and adsorption properties. The incorporation of hydrophilic nanoadsorbent materials into ultrafiltration membranes can improve both the outer surface of the membrane and the internal pore walls, therefore enhancing the antifouling and adsorption performance.

## 5. Future Trends and Challenges for Adsorptive Ultrafiltration Membranes

Generally, compared with nanofiltration and reverse osmosis, adsorptive ultrafiltration membranes are chosen because of their low cost, low-pressure requirement, and high water yield. It was pointed out that the incorporation of adsorbent materials could enhance the performance of the membrane by increasing the functional adsorption sites. Meanwhile, adding hydrophilic adsorbent particles can improve the water flux efficiently and can increase the antifouling properties significantly [51]. However, particle loading is limited due to the affinity between particles and polymers as the low affinity between them leads to poor compatibility. Excessive particle loading will form macropores, which will damage the ultrafiltration performance due to particle aggregation. The agglomeration and low affinity between the adsorbent materials makes it difficult for them to achieve uniform dispersions in the adsorptive ultrafiltration MMMs. Therefore, it is necessary to develop new preparation methods and new adsorbent materials in order to improve the compatibility between the adsorbent and ultrafiltration membranes.

It is also of great importance to use new adsorbent materials with large adsorptive capacity of adsorptive ultrafiltration MMMs for pollutants. Some new adsorbents have excellent adsorptive capacity. For instance, the adsorption capacity of Cu(I)-tpp@ZIF-8 heterostructure adsorbent for P-arsanilic acid is 303.0 mg g^−1^ [52]. The maximum adsorption capacity of as-made CAU-17 for phosphate is up to 216.07 mg g^−1^ [53]. At the same time, in order to enhance the affinity between the adsorbent materials and polymer substrates, new preparation techniques of adsorptive ultrafiltration MMMs have been developed. Zhao [54] prepared strong affinity hybrid membranes via the ex situ layer by layer self-assembly method using gelatin (GE) and GO, which was alternately deposited on hydrolyzed polyacrylonitrile (H-PAN) ultrafiltration membranes through multiple interactions. Researchers also adopt MOF polymer suspension to simultaneously spray self-assembly [55] or MOF interface synthesis [56] and use physical or chemical modification of MOF particles in order to enhance the affinity with the polymer matrix [57,58,59]. Thus, we can use the above methods as a reference in order to prepare adsorptive ultrafiltration MMMs without obvious interface defects.

## 6. Conclusions

The composition of pollutants in wastewater is often very complex and each pollutant has its own different characteristics. At present, multi-unit combination is mainly used to separate pollutants from wastewater, however, their application is limited due to complex operation and high cost. Adsorption ultrafiltration MMMs are a new technology that are used in order to improve the efficiency of multicomponent wastewater treatment, which has been developed to explore their practical application potential. The remarkable advantage of adsorption ultrafiltration technology is that it can complete ultrafiltration and adsorption functions in one unit, remove different kinds of pollutants, or remove one pollutant through deep treatment.

However, there are still many problems and challenges in the application of adsorptive ultrafiltration membranes. For instance, the treatment efficiency of adsorption function and ultrafiltration usually does not work together, as well as this, it is also difficult to scale them up to industrial applications. Therefore, future research should focus on overcoming these challenges in order to make effective use of adsorptive ultrafiltration membranes in the field of water treatment.

## Data Availability

Data sharing not applicable.
